# Assessment of combined antiviral drug treatment against selected α-, β-, and γ-herpesviruses

**DOI:** 10.1186/s12985-026-03303-1

**Published:** 2026-09-22

**Authors:** Lucio Fortelny, Christina Wangen, Debora Obergfäll, Kishore Dhotre, Friedrich Hahn, Julia Tillmanns, Manfred Marschall

**Affiliations:** 1 Harald zur Hausen Institute of Virology, Friedrich-Alexander-Universität Erlangen-Nürnberg (FAU), Erlangen, Germany; 2 Present Address: Institute of Virology, Ulm University Medical Center, Ulm, Germany

**Keywords:** Herpesviruses, Antiviral drugs, Reporter-based analyses, Efficacy and characteristics of selected drugs, Combinatorial treatment, Drug-drug synergies

## Abstract

Human herpesvirus infections cover a wide range of pathogenic outcomes and challenging situations in the medical field. All herpesviral α-, β-, and *γ*-subfamilies comprise representatives with a huge clinical impact on human health. The basic and translational research efforts are strongly directed towards the improved molecular understanding of infections and development of novel antiviral test systems and preventive measures. We utilized cell culture-based quantitative models for the assessment of antiviral prototype drugs against human and animal pathogenic herpesviruses. Our analysis focused on approved, developmental, and experimental compounds. All three investigated herpesviruses showed sensitivity against the selected antivirals. The single-drug antiviral treatments identified mean effective concentrations in the micromolar range, for abemaciclib (ABE) and the pharmacophore compound 020 (P-020), as well as submicromolar to nanomolar range, for brincidofovir (BCV) and cyclin-dependent kinase inhibitor of CDK7 (LDC4297). Time-of-addition experiments addressed questions of temporal potency of inhibition and effects on the expression of individual viral proteins. The results demonstrated that these drugs were mostly robust and did generally show moderate or no difference in antiviral activity when administered pre-, post-, or total duration-infection. By addressing the question of potential drug–drug synergies in combinatorial cotreatment settings, we identified effective conditions of combined antiherpesviral drug treatment (cAHT), such as ABE + LDC4297, as demonstrated for all three viruses. Thus, the study provides new information about antiherpesviral potency and characteristics of a selection of four antiviral prototype inhibitors. Especially, examples of broadness in synergistic combination treatment suggested options to improve antiherpesviral therapy strategies.

**Supplementary Information:**

The online version contains supplementary material available at https://doi.org/10.1186/s12985-026-03303-1.

## Introduction

Human pathogenic species of the family *Herpesviridae* cause lifelong infections, with long periods of latency and clinical quiescence. The primary acute situation of infection, or intermittent phases of viral reactivation, can become life-threatening especially in immunocompromised individuals. The α-, β-, and γ-subfamily type species, and clinically highly important pathogens [1], are represented by herpes simplex virus 1 (HSV-1), human cytomegalovirus (HCMV), and Epstein-Barr virus (EBV) [2–4]. HSV-1 causes painful gingivostomatitis, and, in severe cases, even life-threatening HSV encephalitis (HSE) [5]. HCMV infections lead to very severe forms of virus-associated pneumonia or colitis. Importantly, immunosuppressed patients, patients undergoing chemotherapy, as well as the unborn and neonates, are particularly susceptible to these infections [6]. Especially congenital HCMV infection (cCMV) can lead to microcephaly, sensorineural hearing loss, as well as motor and mental developmental disorders [7]. EBV is commonly associated with infectious mononucleosis, as well as a number of lymphocyte- or epithelial cell-specific malignancies, including Burkitt lymphoma and nasopharyngeal carcinoma [8]. Numerous reports illustrate the significant clinical impact of these widespread human viral infections, for which options of therapy and vaccination are still limited [9]. The main issues are side effects of antiviral drugs [10, 11], the development of antiviral resistance, or a lack of broad-spectrum antiherpesviral efficacy of treatment options [12]. In this regard, the identification of new accessible targets of antiviral therapy and the development of improved therapeutic strategies are urgently required.

Previously, we described pharmacological inhibitors of cyclin-dependent kinases (CDKs), which comprised high potency as experimental antiviral small molecules against HCMV [13–15] and, in part, also against a spectrum of other herpesviruses [16, 17]. A combination of host-directed antivirals (HDAs), such as CDK inhibitors, and direct-acting antivirals (DAAs), such as inhibitors of the HCMV-encoded CDK ortholog pUL97/vCDK, produced strong synergistic effects in vitro and in vivo [14, 18]. Abemaciclib (ABE), a prominent CDK4/6 inhibitor, was originally developed for the treatment of hormone receptor (HR)-positive, human epidermal growth factor receptor 2 (HER2)-negative breast cancer [19], and has been approved for clinical use in oncology [20]. In addition to its use in tumor therapy, its antiviral efficacy, primarily against HCMV infection of primary human fibroblasts and murine cytomegalovirus (MCMV) infection in a murine animal model, has recently been demonstrated [15]. The main mechanistic basis of the antiviral activity of ABE against HCMV is due to the functional role of CDK4/6 in the viral productive replication cycle. This may include a virus-supportive regulation of gene expression as well as a CDK-mediated phosphorylation of viral proteins, as this has likewise been shown for CDK7 [21]. Moreover, the antiviral activity of ABE may also reach beyond its CDK4/6-specific activity, referring to the recent finding that ABE suppresses the proviral phosphorylation of SAMHD1 by pUL97 [13, 22]. This suggested dual function of ABE, in both CDK4/6-directed and CDK4/6-independent aspects, may give rise to potential synergistic combination treatments using ABE, and additionally to combinations between other HDAs and DAAs.

Building on previous findings, focusing on investigational treatment approaches with combined antiviral kinase inhibitors, we now studied potential examples of broadly acting drug synergisms directed against α-, β-, and γ-herpesviruses. For this approach, we selected four clinically relevant drugs, which represented different levels of compound analysis, namely approved (ABE, breast cancer treatment), developmental (brincidofovir/BCV, a putative broad-spectrum antiherpesviral; LDC4297, a highly selective CDK7 inhibitor with antitumoral activity), as well as an experimental drug (P-020, a pharmacophore-derived antiherpesviral prototype). The distinct antiviral mechanisms of action have been described recently (Fig. S1), such as the inhibitory effects directed against host CDKs (ABE, LDC4297), the viral DNA polymerase (BCV), or the viral nuclear egress complex (P-020). We initially performed single-drug antiviral treatments with these compounds to identify both, specific hits and broad antiviral activities. Drug combinations between host-directed and direct-acting prototypes, HDAs + DAAs, were then examined using the established method of Loewe additivity fixed-dose assay. Mechanistic aspects of antiviral drug activity were addressed by time-of-addition experimentation. A comparison between prophylactic and therapeutic drug administration (i.e. treatment start prior or post infection, respectively) was finally performed. In essence, our data support novel options of broad-spectrum intervention, based on the approach of combined antiherpesviral drug treatment (cAHT).

## Methods

### Cell culture and viruses

Primary and immortalized cell types of human or animal origin were cultivated under standard conditions published elsewhere [23]. The viruses used were herpes simplex virus type 1 expressing the green fluorescent protein reporter (HSV-1 VP22-GFP; [24]), human cytomegalovirus strain AD169 expressing the green fluorescent protein reporter (HCMV AD169-GFP; [25]), and murine herpesvirus 68 strain myodes expressing the firefly luciferase reporter [26]; for additional details, see Supplementary Information.

### Antiviral compounds

Antiviral compounds used in this study were the two clinically relevant drugs abemaciclib (ABE) and brincidofovir (BCV), as well as the experimental drugs LDC4297 (selective CDK7 inhibitor) and P-020 (pharmacophore-derived NEC-binding compound with nuclear egress-inhibiting activity) as described previously [15, 17, 23]; for details and the sources of compounds, see Supplementary Information.

### Cytotoxicity assessment via Neutral Red (NR) uptake assay

To determine cell viability in a quantitative manner, the NR uptake assay was performed as described previously [23, 27]. In brief, 1.35 × 104 cells/well were seeded in a 96-well plate and cultivated overnight, before treatment with compounds, solvent control DMSO, or 1 μM staurosporine as a cytotoxic positive control, was performed for a duration of 7 d. NR solution (40 μg/mL) was added and cellular NR uptake was quantified using a Victor X4 Multilabel Microplate Reader (PerkinElmer, Waltham, MA, USA; 560/630 nm); for details, see Supplementary Information.

### Assessment of viral replication via fluorescence- and luminescence-based reporter readouts

To assess the antiviral potency of compounds, we used reporter protein-based virus replication assays as published elsewhere [23, 27–30]; For HSV-1 and HCMV, 1.35 × 104 HFFs/well were seeded in a 96-well plate, and infection was performed with HSV-1 VP22-GFP or HCMV AD169-GFP at MOI of 0.25-0.5 GFP-forming units (FU)/7 d. For MHV-68, the luciferase-based system using MHV-68-Luc was employed, with 3 × 104 Vero cells/well seeded in a 96-well format and infected at MOI of 0.25-0.5 Luc-FU/4 d. At the time points of 32 h p.i (HSV-1), 7 d p.i. (HCMV), or 4 d p.i. (MHV-68), cells were processed for quantitative measurement of the viral GFP or Luc reporter signal using a Victor X4 microplate reader (PerkinElmer); for details, see Supplementary Information.

### Compound interaction analysis via Loewe additivity fixed-dose and Bliss independence checkerboard assays

The protocols of Loewe additivity fixed-dose assay and Bliss independence checkerboard assay, as adapted to various conditions of virus infection, have been described before [14, 15, 18]; For this, we pre-determined the effective concentrations of antiviral compounds analyzed in single treatment, and then performed serial dilutions of compound cotreatment to cover the biologically relevant ranges of activity. In this study, the analysis of compound interactions was primarily based on the Loewe assay, in which antiviral efficacy was determined using CompuSyn software for quantitation of the combination index (CI). The subsequently calculated weighted CI (CIwt) allowed to interpret the interaction pattern of synergistic, additive, or antagonistic; for details, see Supplementary Information.

### Time-of-addition (ToA) antiviral drug analysis

The conditions of ToA antiviral drug analysis were described in the context of Fig. 2; In brief, a putative impact of the timing of compound addition was assessed by applying differential temporal anti-HCMV treatment conditions of the drugs ABE, BCV, LDC4297, and P-020, for eventually measuring the viral reporter GFP signal in dependence of drug concentration and ToA; for additional details, see Supplementary Information.

## Results and discussion

### Selected herpesviruses for comparative antiviral drug analysis

**Fig. 1:**
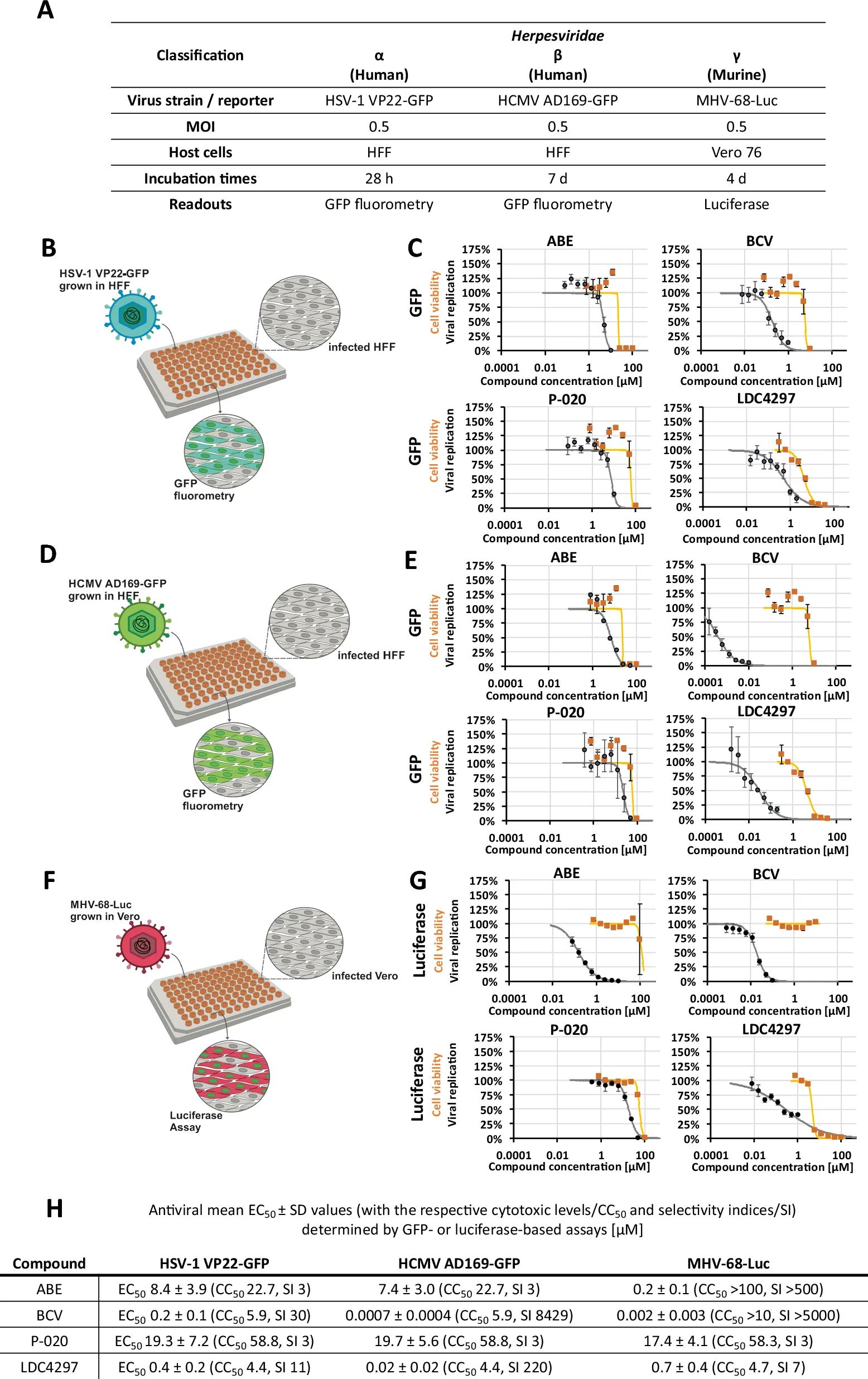
Assessment of antiviral activity and cell viability of direct-acting and host-directed antivirals against selected herpesviruses. (**A**) Overview of the utilization of our recently established antiherpesviral assay systems. The scheme illustrates the respective parameters of three reporter-based assay systems: (**B**) HSV-1 VP22-GFP infection in primary HFF cultures, (**D**) HCMV AD169-GFP in HFFs, and (**F**) MHV-68-Luc in immortalized Vero 76 cells. (**C**, **E**, **G**) Antiviral activity of the four analyzed compounds, ABE, BCV, P-020, and LDC4297, was demonstrated in a comparative setting, using HSV-1 (28 h p.i.), HCMV (7 d p.i.), and MHV-68 (4 d p.i.), respectively. Cell viability was assessed via a Neutral Red uptake assay at the identical time points. Results are shown as percentages relative to the solvent control (DMSO). (**H**) Summary of antiherpesviral EC_50_ values of the mechanistically different prototype drugs ABE, BCV, P-020, and LDC4297. Values were based on n=2 independent experiments, each based on measurements in quadruplicate (antiviral assays) or triplicates (Neutral Red assay)

In order to address the medical importance of α-, β-, and *γ*-herpesviruses, in particular through antiviral drug research [28–30], we have continuously focused on the establishment of quantitative antiviral drug assessment systems. These were based on various reporter and readout technologies, as applied for herpes simplex virus 1 (HSV-1), varicella zoster virus (VZV), human cytomegalovirus (HCMV), Epstein-Barr virus (EBV), its animal homolog, murine herpesvirus 68 (MHV-68), and several more [14–17, 23, 27, 31–34]. In particular, the MHV-68-Luc attracted our interest, because it undergoes a productive lytic replication in several permissive cell types and can be used as a reporter-expressing model. In that way, MHV-68-Luc represents a well-characterized homolog of EBV/*γ*-herpesviruses, and has thus been adapted to our quantitative system of antiviral drug assessment very recently (Wangen et al., 2026, Virus Res., under revision). One current finding resulted from the comparison of cell types in regard of their permissiveness for productive MHV-68-Luc replication, i.e. indicating full permissiveness of Vero and COS-7 cells as well as primary murine fibroblasts MEFs, whereas human HFFs showed a very low permissiveness. For the quantitative settings of antiviral drug assessment, Vero 76 cells comprised highest sensitivity and reliability (Fig. S1; for methodological details see [23]). Next, comparing three reference herpesviruses, we addressed the following points in quantitative and qualitative terms: (i) the potency of single-compound antiviral treatments using approved, developmental, and experimental drugs, (ii) the potential occurrence of drug–drug synergies in combinatorial cotreatments, (iii) the broadness of drug potency and synergy, as compared between the selected α-, β-, *γ*-herpesviruses, and (iv) the characterization of mechanistic details of antiviral drug activities, especially addressed by antiviral time-of-addition experimentation. The applied systems of antiviral drug assessment spanned species taken from the herpesvirus subfamilies, several readout and reporter measurements, and primary, as well as immortalized cell types (Fig. 1A). Based on this methodological platform, a comparative analysis of antiviral compounds was performed. These included a selection of both direct-acting (DAAs) and host-directed antivirals (HDAs).

### Evaluation of approved drugs, developmental candidates, and experimental compounds, in settings of single-drug antiviral treatment

Recently, we demonstrated the antiviral potency of a variety of CDK inhibitors and showed that many of them exhibited pronounced antiviral activities, such as anti-HCMV, in the nanomolar range [14, 17, 18]. Here, four selected compounds were characterized by antiviral parallel settings, and data were correlated with cytotoxicity levels, i.e. abemaciclib (ABE, targeting host CDK4/6), brincidofovir (BCV, targeting viral polymerase), P-020 (targeting viral nuclear egress), and LDC4297 (targeting host CDK7; Fig. S2). These drugs were analyzed against HCMV, HSV-1, and MHV-68, using human and animal cell types in cultured-cell infection models (Fig. 1B, D, F). It should be emphasized that MHV-68 replication can be analyzed in various cell types (murine, simian, human cells; [23]), and that our comparative analyses indicated most accurate quantitative evaluation achieved in Vero 76 cells. In all cases of infection with the three viruses, a concentration-dependent inhibition of viral replication could be observed (Fig. 1C, E, G), whereby the separation from cytotoxic drug concentrations was very pronounced in some cases (e.g. BCV, LDC4297), but moderate in others (e.g. ABE, P-020). Hereby, the time point of measuring cytotoxicity (Fig. 1A-H) was identical with the measurement of antiviral activities (HSV-1, HCMV, MHV-68). It should be emphasized that an additional microscopic inspection was routinely done throughout the treatment period, to monitor the putative appearance of morphologically detectable changes in cell viability. This visual monitoring did not reveal signs of drug-mediated effects, whereby this additional control, however, could not fully exclude any later onset of cytotoxicity (> 7 d). The mean values of antiviral effective half-maximal drug concentrations (EC_50_) were ranging in micromolar (ABE, P-020), submicromolar (LDC4297), to nanomolar/subnanomolar concentrations (BCV). Specifically strong antiviral potency was found against HCMV (EC_50_ values between 0.0007 ± 0.0004 µM and 19.7 ± 5.6 µM), as well as some lower potency against the other two viruses, HSV-1 and MHV-68 (EC_50_ values between 0.4 ± 0.2 µM and 19.3 ± 7.2 µM, or 0.002 ± 0.003 µM and 17.4 ± 4.1 µM, respectively; Fig. 1H). Concerning a putative interference of cytotoxicity, three of the four compounds, BCV, LDC4297, ABE, had been well-characterized by previous reports [15, 17, 35], while the relatively new pharmacophore compound 020 (P-020) was now additionally analyzed for cytotoxicity levels in several different cells types ([36]; Fig. S3). Of note, for the five selected cell types, the onset of main P-020 cytotoxicity was in the concentration range of 25–100 µM, thus was found distinct from the effective antiviral concentrations. These findings illustrate the broadness of antiherpesviral activity of these drugs, albeit at differential quantitative levels.

### Antiviral drug time-of-addition experiments: characterization of the individual inhibitory properties

**Fig. 2:**
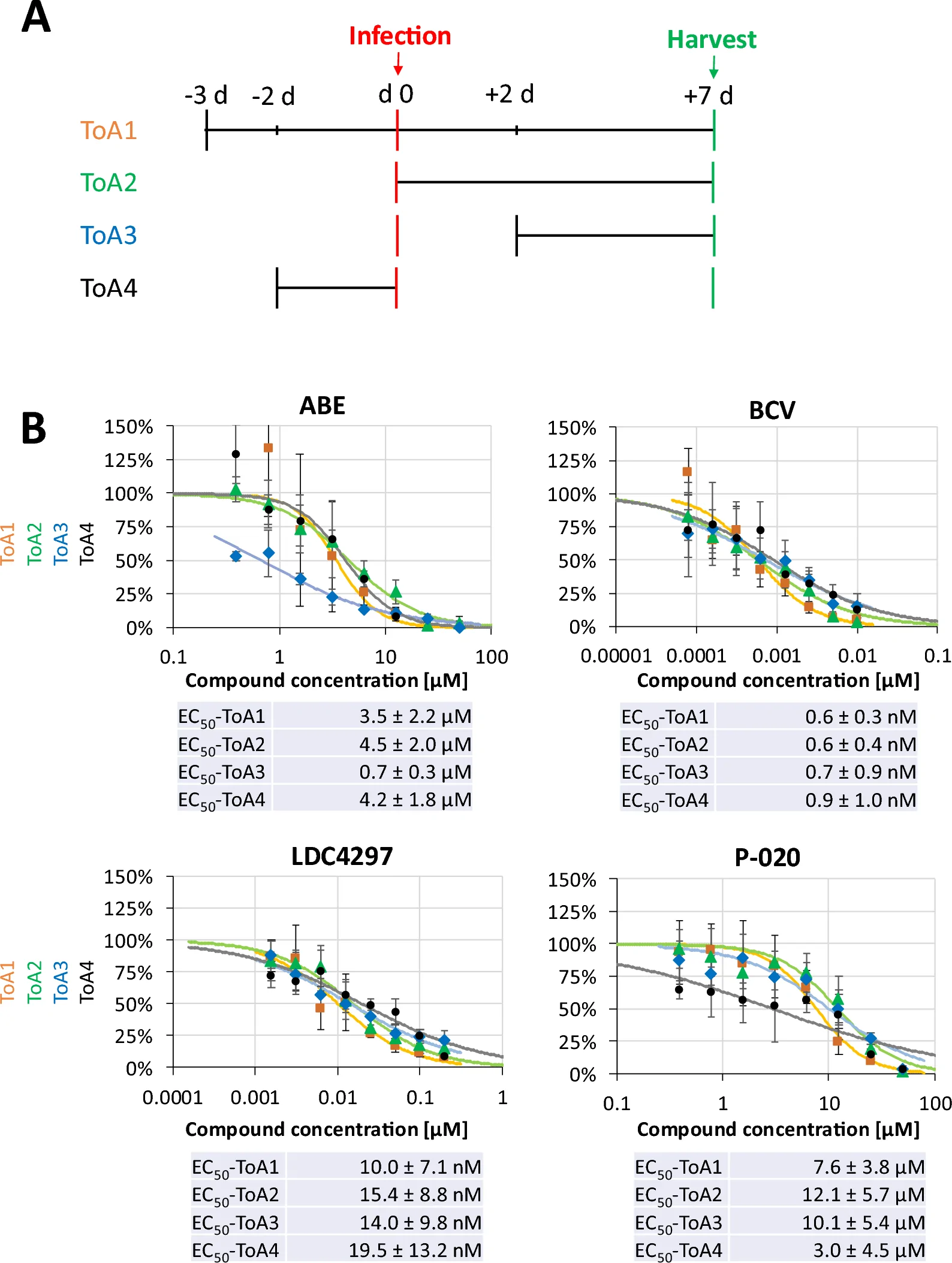
Analysis of time dependency of the antiviral drug activity against HCMV. (**A**) HFFs were infected with HCMV AD169-GFP for 7 d (MOI 0.25–0.5 GFP-FU/7d). Antiviral compounds (ABE, BCV, P-020, LDC4297) were added at different time points as indicated: Pretreatment − 3 d until harvest +7 d (time-of-addition schedule, ToA1), addition simultaneously with virus inoculum d 0 (ToA2), delayed onset of treatment +2 d (ToA3), or prophylactic treatment only − 2 d to d 0 (ToA4). (**B**) HFFs were harvested 7 d p.i., to determine the antiviral activity via quantitation of the viral GFP reporter signal. The investigation included n=2 independent experiments, whereby EC_50_ values were given as mean values of quadruplicates ± SD (note that values for ABE and P-020 are in the µM unit, while BCV and LDC4297 are in the nM unit)

**Fig. 3:**
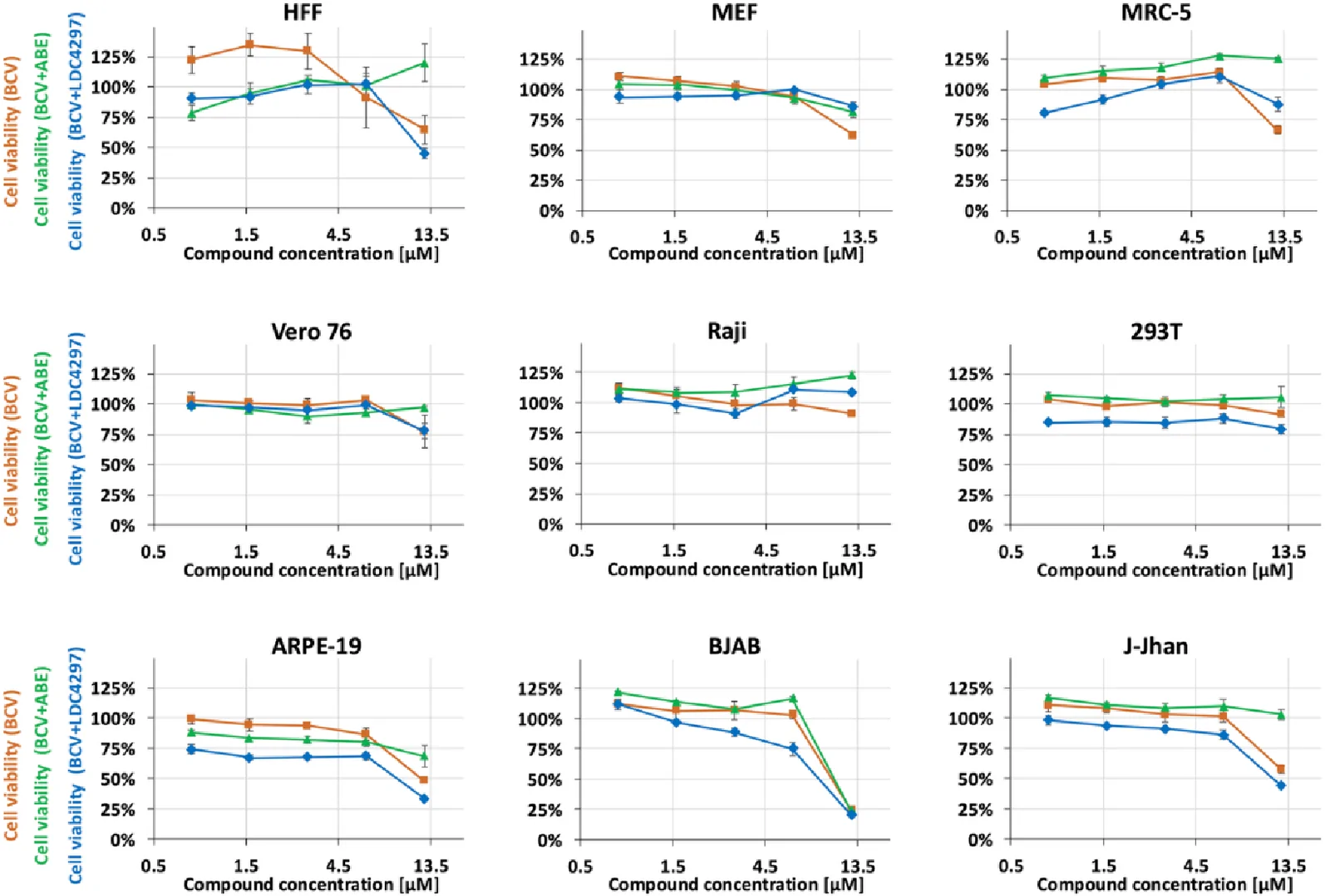
Experimental setup that argues against a putative enhancement of cytotoxicity through combined drug-drug cotreatment. Selected cell types were treated either with BCV alone (BCV; orange curves), at a concentration panel of 12.5, 6.3, 3.1, and 1.6 μM, or with a combination of BCV plus 1 μM ABE (BCV + ABE; green curves) or plus 1 μM LDC4297 (BCV + LDC4297; blue curves). The cell types comprised a spectrum of human primary cells (HFF, MEF), immortalized cell lines (293 T, ARPE−19, MRC-5), suspension cells (Raji, BJAB, J-Jhan), and a simian cell line (Vero 76). At 5 d post-treatment, cell viability was measured by Neutral Red uptake assay (triplicate determination), and mean values ± SD are expressed as a percentage relative to DMSO control. The experiments were independently reproduced (n=2, see also Fig. S3)

**Fig. 4:**
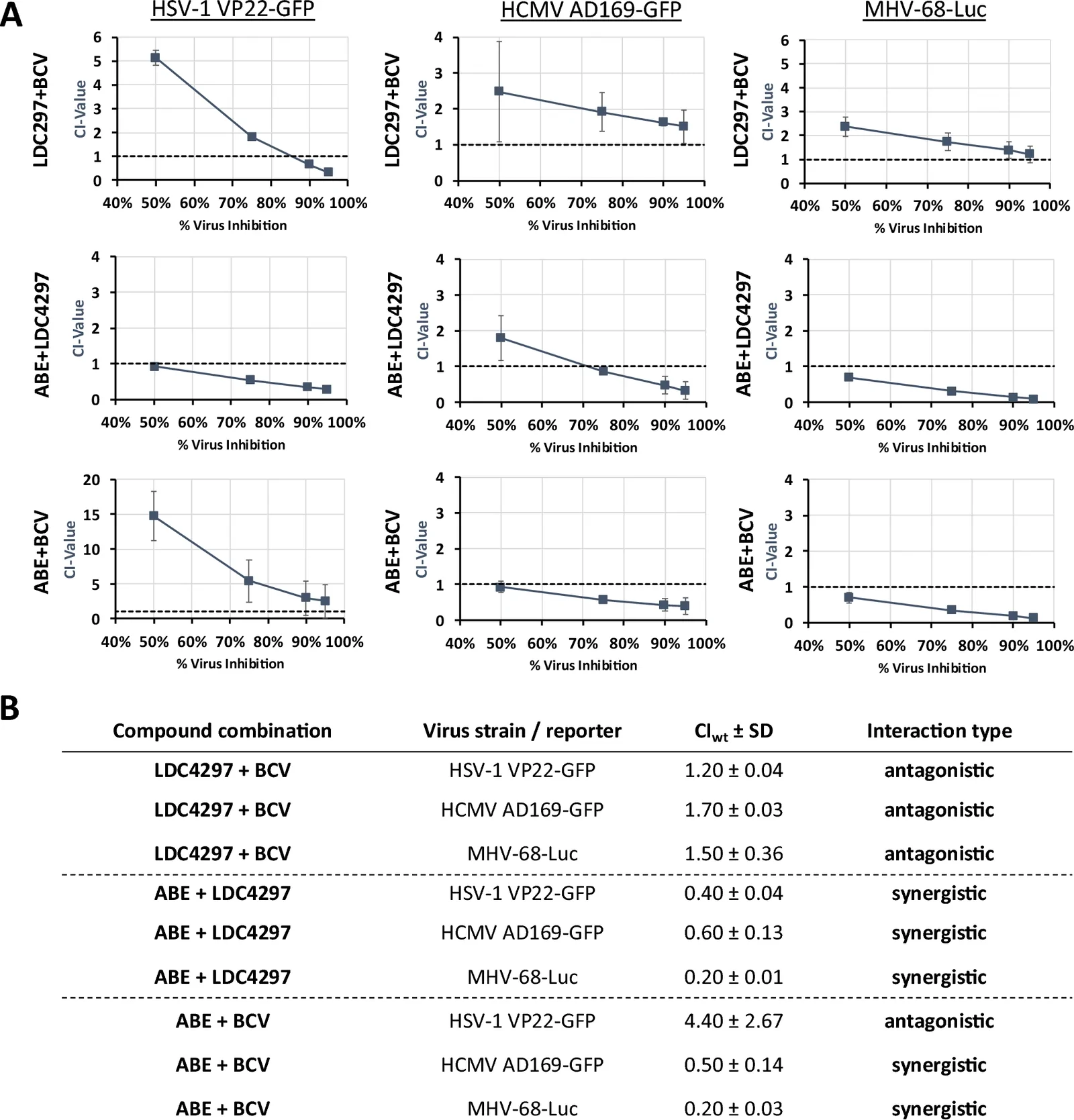
Combinatorial drug-drug cotreatment determined by Loewe additivity fixed-dose assay. (**A**) The three drug combinations of ABE + BCV, ABE + LDC4297, and LDC4297 + BCV were determined in the antiviral assay systems against HSV-1 VP22-GFP, HCMV AD169-GFP, and MHV-68-Luc. Detailed ratios of drug combinations were as follows: ABE + BCV (4:1) for HSV-1, (418:1) for HCMV, and (11:1) for MHV-68, respectively; ABE + LDC4297 (4:1) for HSV-1, (101:1) for HCMV, and (1:3.5) for MHV-68, respectively; LDC4297 + BCV (2:1) for HSV-1, (5:1) for HCMV, and (26:1) for MHV-68, respectively. The selection of distinct drug combination ratios has been based on the individual pretesting of several comparative concentration settings, to identify the most appropriate ratios, as described before [15, 18]. The CI_wt_ values, defining the drug interaction type, were calculated as (1 × CI_50_ + 2 × CI_75_ + 3 × CI_90_ + 4 × CI_95_)/10. The types of drug interaction were set as follows: values 0 to <0.9, synergistic; 0.9 to 1.1, additive (denoted by dotted line at CI = 1); >1.1 to ≥4.4 antagonistic [15]). (**B**) Final results of the quantitative drug-drug interaction analysis: Data are presented as mean weighted CI values (CI_wt_ ± SD), corresponding to the defined values of virus inhibition (50%, 75%, 90%, 95%), collected from n=2 independent experiments

In order to characterize the anti-HCMV properties of the analyzed drugs in more detail, a time-of-addition (ToA) experiment was performed (Fig. 2). To this end, four schemes of drug addition were applied (Fig. 2A): ToA1 (pretreatment starting 3 days prior to infection, continued until harvest at d 7), ToA2 (treatment added simultaneously with virus inoculum at d 0, continued until harvest), ToA3, (delayed addition +2 d), or ToA4 (exclusively added as pretreatment/prophylaxis − 2 d to d 0). These experiments, like other antiviral assessments, were performed at low MOI, in order to avoid a saturation level of virus production (that might prevent the recognition of drug inhibitory effects). This determination of inhibitory potencies, in the drug concentration-dependent manner, further illustrated the anti-HCMV potency of all four analyzed compounds. Antiviral potency was measured at 7 d p.i. which represented the inhibition of viral replication within the third replicative round (approx. 3 d/round, in the case of HCMV AD169-GFP). The quantitative evaluation was expressed by the EC_50_ values given below each panel (Fig. 2B). Notably, the antiviral potency of these compounds appeared rather stable under the four ToA conditions applied. One exception was provided by ABE treatment, under delayed addition of ToA3 (+ 2 d; blue curve), indicating enhanced activity compared to the other three schemes of ABE addition. This may indicate the relevance of a peak level of ABE treatment at an early time of infection. Interestingly, the scheme of exclusive pretreatment/prophylaxis (− 2 d to d 0) showed strong anti-HCMV for three of the four compounds, i.e. ABE, BCV, and LDC4297. This may indicate a rapid drug uptake into HFFs, and a consistent inhibitory activity in the multi-round virus replication setting. Notably, BCV comprises a well-known durability in cells (i.e. an effectivity ≥100 h upon single treatment; [37]). Here, another exception was provided by P-020, which has been characterized as a compound that blocks viral nuclear egress [32], thus representing a late-phase inhibitor of HCMV replication. According to these ToA schemes, the presence of P-020 during the early or late time points of infection was obviously similarly effective (including certain degree of variability). Of note, especially the very early presence of the drug (− 3 d) or exclusive pretreatment in ToA1 or ToA4, respectively, provided high antiviral activity, with the respective lowest EC_50_ values among the four conditions. This points to a relatively high stability of P-020 and possibly intranuclear accumulation over time. It has to be explained that P-020 represents a newly identified antiherpesviral prototype drug [31], which will be described in detail in a separate report (Marschall, Tillmanns et al., manuscript in preparation), and respective data were therefore not included in the combination experiments described below (Figs. 3 and 4). It should additionally be noted that, within this multi-round replication setting, viral IE, E and L genes are progressively expressed (cascade-like within the first round, more agitated patterns of expression in next rounds), and viral DNA is also advancing during the replication time. This represents a general limitation of TOA studies, which are employed in many antiviral drug research approaches, thus making a clear-cut mechanistic conclusion demanding (including the question of a drug’s effective period; [38]). Nevertheless, these results confirmed the robust anti-HCMV activity profiles of the four compounds across the ToA1-4 conditions analyzed.

### Addressing the potential drug–drug synergies in combinatorial cotreatment settings

To analyze the combined drug treatment, we first wished to rule out the possibility of a mutually reinforced cytotoxicity. To this end, we performed a scheme of several cotreatments between the drugs BCV, ABE, and LDC4297 (Fig. 3). Hereby, we used nine different cell types, including primary or passage-extended human culture cells (HFF, MEF, MRC-5), immortalized monolayer cells (Vero 76, 293 T, ARPE−19), and immortalized suspension cells (Raji, BJAB, J-Jhan). As a result, some minor variability in drug-induced effects was observed between the selected cell types, but in no case any marked enhancement of cytotoxicity could be identified. This indicated that cytotoxicity of cotreatments, at least under the chosen experimental conditions for combined exposure, remained in an expected range. Thus, regarding the subsequent antiviral drug–drug interaction analyses, an indirect impact due to combinatorial enhancement of cytotoxicity could therefore be made rather improbable.

In previous studies, we demonstrated drug–drug combinations with strong antiviral synergy against HCMV [15]. Here, we intended to expand this spectrum to include HSV-1 [24] and the murine EBV homolog MHV-68 [26]. The selected experimental combination treatment consisted of the single components ABE, LDC4297 and BCV. In our main point of interest, we used the Loewe additivity fixed-dose assay, to assess drug interaction types by calculating Combination Indices (CI) (Fig. 4). Interestingly, the combination of LDC4297 + BCV exhibited an antagonistic effect in the three examples analyzed (Fig. 4A, upper panels). This may be explained by the fact that developmental drug BCV acts as an inhibitor of the HCMV polymerase complex, which additionally requires the activity of phosphorylating kinase activity such as cyclin-dependent kinase CDK7 [14]. An alternative explanation for the antagonism of LDC4297 + BCV treatment may be given in the way that LDC4297 inhibits CDK7, which is involved in transcription initiation, thereby potentially inhibiting the expression of the viral DNA polymerase and accessory early proteins possibly targeted by BCV. Thus, we hypothesize that the CDK7 inhibition by LDC4297 can explain the observed antagonistic effect in combination with polymerase inhibitor BCV for HCMV, and possibly in a similar way for HSV-1 and MHV-68. In contrast to this finding, a true synergistic effect was observed with the combination ABE + LDC4297 for HCMV, which was consistent with an earlier report [15]. Moreover, the data of our present study also demonstrated this synergistic effect for HSV-1 and MHV-68 (Fig. 4A, middle panels). This may be explained by a broad herpesviral replication-supportive effect of the two host kinases CDK4/6 and CDK7, which may be mechanistically realized through two different effector events (i.e. possibly CDK4/6 in promoting the cell cycle G1/S transition, and CDK7 in the onset of transcription), so that the combination of the respective inhibitory effects results in a synergism. Finally, the combination of ABE + BCV showed synergistic effects for both HCMV and MHV-68, whereas this was not observed for HSV-1, but in contrast, an antagonism was measured (Fig. 4A, lower panels). Thus, we hypothesized that the combined targeting of CDK4/6 (by ABE), together with the viral polymerase (by BCV), was critical for the replication of HCMV and MHV-68, but less critical for HSV-1. In the case of HSV-1, the combinatorial inhibition of the host CDK4/6 and viral polymerase showed an even antagonistic result, which may indicate a so far unknown regulatory interplay between these two targets, in the case of α-herpesvirus, but not the compared β- and *γ*-herpesviruses. Although the underlying mechanistic basis of this observation remains unresolved so far, the antagonistic effect was found independent from the assay system. In the case of ABE + BCV combination against HSV-1 replication, we performed a confirmative analysis by the use of the Bliss independence checkerboard assay in parallel. Also, these results, evaluated with MacSynergy II software [39], indicated an antagonistic effect between these two drugs (Fig. S4). A synopsis of findings was listed below (Fig. 4B), showing the CI_wt_ values of 1.20 ± 0.04, 1.70 ± 0.03, 1.50 ± 0.36 (LDC4297 + BCV), and 0.40 ± 0.04, 0.60 ± 0.13, 0.20 ± 0.01 (ABE + LDC4297), and 4.40 ± 2.67, 0.50 ± 0.14, 0.20 ± 0.03 (ABE + BCV) for HSV-1, HCMV, and MHV-68, respectively. In essence, our results on combinatorial drug treatments analyzed for three herpesviruses identified those synergistic cotreatment effects, suggesting further elaboration towards effective antiherpesviral targeting strategies.

## Conclusions

This study focused on a quantitative comparison of the antiviral potency of approved drugs (ABE), developmental candidates (BCV), and experimental prototypes (LDC4297, P-020). For three selected members of α-, β-, and γ-herpesviruses, an initial single drug analysis was then extended with an assessment of drug–drug interactions. Our previous studies have been directed to similar investigations for the β-herpesviruses HCMV and MCMV [15], so that here, a broadening of information was intended for further herpesviruses undergoing productive replication in cultured cell systems [16, 23, 33]. The main outcomes of our study were as follows: (i) four compounds of differential developmental stages were comparatively analyzed, i.e. abemaciclib (ABE; [15]), brincidofovir (BCV; [35]), the NEC-directed experimental drug 020 (P-020; [31]), and a highly selective CDK7 inhibitor (LDC4297; [17]); (ii) this compound selection possessed different targets and mechanistic features, and covered both direct-acting antivirals (DAAs) and host-directed antivirals (HDAs); (iii) all compounds exhibited a concentration-dependent antiviral activity against the herpesviruses HSV-1, HCMV, and MHV-68; (iv) in time-of-addition experiments, the drugs were further characterized under variable conditions of drug exposure and showed robust antiviral potency patterns; (v) finally, a combinatorial cotreatment analysis (Loewe additivity fixed-dose assay), revealed virus-specific effects, thus differentiating between synergistic and antagonistic drug-drug interactions. Importantly, the identified combination of ABE + LDC4297 displayed a synergistic antiviral effect against the selected α-, β-, and γ-herpesviruses. Of note, a confirmed absence of relevant reinforced cytotoxicity levels, measured under combinatorial drug exposure conditions, may underscore their use in potential developmental processes. In essence, the experimental approaches identified drug combinations that exhibit a strong synergistic antiviral activity. The findings may also suggest a broader-ranging antiherpesviral cotargeting strategy. These might include both DAAs and HDAs, possibly offering chances to enhance antiviral potency through the restriction of drug concentrations to low-dosage ranges, without a proportional increase of cytotoxic effects or antiviral drug resistance. The clinical relevance of such identified drug combinations is still in question, since there are numerous examples of synergistic combinations measured in cultured cells models, which did not translate into clinical utility. Nevertheless, there can be no doubt that novel antiviral combination treatments are urgently requested by various clinical situations. Especially, the resistance-suppressing combinations between HDA plus DAA drugs identified in our study appear promising (e.g. ABE + BCV shown for the model of *γ*-herpesvirus infection). The latter example might offer new chances in *γ*-herpesvirus treatment options, such as the unresolved issue of Epstein-Barr virus-associated post-transplant lymphoproliferative disorder (PTLD). ABE represents a clinically approved cancer drug [20] and BCV showed antiherpesviral potential in previous trials of drug development [40]. These combinatorial aspects may be of specific relevance for immunocompromised patients, and other cases, in which monotherapy is frequently insufficient or dose-limited by toxicity. In essence, such novel drug targeting options may mostly aim at HCMV-specific and EBV-specific clinical risk situations. Combined, our findings suggest that further research should follow to focus on mechanistic investigations, to clarify molecular mechanisms, to ascertain the qualities of synergistic interactions, and to foster the development of antiviral treatment opportunities.

## Supplementary Information

Supplementary Material 1.

## Data Availability

All datasets generated were presented in the current study.
