# Supplementary material for: Assessment of combined antiviral drug treatment against selected α-, β-, and γ-herpesviruses
Source: Virol J. 2026 Sep 22;23:219. doi: 10.1186/s12985-026-03303-1 (PMC13595758; doi:10.1186/s12985-026-03303-1)
Supplement: Supplementary file 1 [file 12985_2026_3303_MOESM1_ESM.pdf]

Virology Journal

BRIEF REPORT Open Access

Supplementary Information

Assessment of combined antiviral drug treatment against selected α-, β-, and γ-herpesviruses

Lucio Fortelny[a], Christina Wangen[a], Debora Obergfäll[a], Kishore Dhotre[a], Friedrich Hahn[a,§], Julia Tillmanns[a], Manfred Marschall[a,*]

[a] Harald zur Hausen Institute of Virology, Friedrich-Alexander University of Erlangen- Nürnberg (FAU), Erlangen, Germany

[§] Present address: Institute of Virology, Ulm University Medical Center, Ulm, Germany

* Corresponding author: manfred.marschall@fau.de

E-mail addresses: lucio.fortelny@fau.de; christina.wangen@uk-erlangen.de; debora.obergfaell@fau.de; kishore.dhotre@uk-erlangen.de; friedrich.hahn@uni-ulm.de; jul.tillmanns@fau.de; manfred.marschall@fau.de

Supplementary Methods

Cell culture and viruses

Primary human foreskin fibroblasts (HFFs) were cultivated in Minimal Essential Medium (MEM, 21090022; Thermo Fisher Scientific, Waltham, MA, USA) supplemented with 10% fetal bovine serum (FBS, F7524; Sigma Aldrich, St. Louis, MO, USA), 1% GlutaMAX™ (35050038, Thermo Fisher Scientific), and 10 µg/mL gentamicin and incubated at 37 °C, 5% CO_2, and 80% humidity. African green monkey kidney cells (Vero 76) were cultivated in Dulbecco's Modified Eagle's Medium (DMEM; Thermo Fisher Scientific) supplemented with 10% FBS, 1% GlutaMAX™, 10 µg/mL gentamicin, and incubated at 37 °C, 5% CO_2, 80% humidity. HFFs were used for infection experiments with HSV-1 VP22-GFP and HCMV AD169-GFP; Vero 76 cells for infection with MHV-68-Luc.

Antiviral compounds

Antiviral compounds were obtained from the following sources: abemaciclib (ABE; MedChemExpress, Monmouth Junction, NJ, USA), brincidofovir (BCV; Biomol GmbH, Hamburg, Germany), LDC4297 (Lead Discovery Center GmbH, Dortmund, Germany) and the NEC-binding experimental drug P-020 (AiCuris AG, Wuppertal, Germany). The compounds were dissolved in sterile DMSO (Sigma Aldrich) and stored at -20 °C.

Cytotoxicity assessment via Neutral Red (NR) uptake assay

To determine the cytotoxicity of the compounds (1), the Neutral Red (NR) uptake assay was performed. For this purpose, 1.35×10^4 cells/well were seeded in a 96-well plate. One d post-seeding, cells were treated with the compound, the solvent control (DMSO), or 1 µM staurosporine as a cytotoxic positive control. At 7 d post-treatment, 40 µg/mL of NR was added and the plates were incubated for approx. 4 h at 37 °C, before removing NR solution and destaining the cells with empty buffer. NR uptake was quantified using a Victor X4 Multilabel Microplate Reader (PerkinElmer, Waltham, MA, USA; 560/630 nm).

Assessment of viral replication via fluorescence- and luminescence-based reporter readouts

To assess the antiviral efficacy of compounds, we used green fluorescence protein (GFP) and luciferase (Luc) based reporter systems, as described previously (2-8). For HSV-1 and HCMV, 1.35×10^4 HFFs/well were seeded in a 96-well plate, and infection was performed with HSV-1 VP22-GFP or HCMV AD169-GFP at MOI of 0.25-0.5 GFP-FU/7 d. For MHV-68, the luciferase-based system using MHV-68-Luc was employed, with 3×10^4 Vero cells/well seeded in a 96-well format and infected at MOI of 0.25-0.5 Luc-FU/4 d. At the time points of 32 h p.i (HSV-1), 7 d p.i. (HCMV), or 4 d p.i. (MHV-68), cells were fixed for quantitative measurement of the viral GFP, or directly used without fixation for Luc reporter signal detection, both applying a Victor X4 microplate reader (PerkinElmer). Alternatively, after fixation, cells were permeabilized using 0.2% Triton-100 and the nuclei were stained using Hoechst dye (Hoechst GmbH, Frankfurt am Main, Germany), before GFP measurement using the ImageXpress Pico (Molecular Devices, LLC, San Jose, CA, USA). For the Luc-based measurements,

cells were lysed using lysis buffer consisting of a 100 mM phosphate buffer, 15 mM $MgSO_4$, and 1% Triton X-100. Then, a volume of 50 µL of lysis buffer, including 10% ATP and 1 mM D-luciferin (PJK, Kleinbittersdorf, Germany), was added per well. The luminescence emission of samples was immediately detected using a Victor X4 Multilabel Microplate Reader (PerkinElmer); for details, see Supplementary Information.

Compound interaction analysis via Loewe additivity fixed-dose assay

The Loewe additivity fixed-dose assay was primarily used in this study for the assessment of drug–drug interaction, aiming at the identification of synergistic antiviral effects and a putative reduction of effective antiviral dosages. For this purpose, we pre-determined effective antiviral concentration ranges of single-drug treatments. Then, selected fixed ratios of antiviral cotreatments were applied in eight-fold serial drug dilutions, incubated on layers of 1.35×10^4 cells/well seeded in a 96-well plate and used for virus infection, to cover the biologically relevant range of activity. The antiviral efficacy was determined using CompuSyn software (9) and was quantified in terms of the combination index (CI) (5, 10, 11). The subsequently calculated weighted CI (CI_{wt}) allowed to interpret the interaction pattern of synergistic, additive, or antagonistic. Only results with an r-value greater than 0.9 were included in the final evaluation.

Compound interaction analysis via Bliss independence checkerboard assay

In addition, the Bliss independence checkerboard assay was used as a confirmation system in specific cases (12). Hereby, serial dilutions of both compounds were applied across a broad matrix of concentration pairs as described previously (6). In brief, 1.35×10^4 cells/well were seeded in a 96-well plate and used for virus infection (e.g. HSV-1, Fig. S4). After the 90-min phase of virus adsoption, inhibitors were added according to an 8x8 matrix, with DMSO as the solvent control, or MEM as empty medium control. For each setting of the experimental replicate(s), technical triplicates of samples were used. At 32 h p.i (HSV-1), cells were fixed and used for the measurement by Victor X4 Multilabel Microplate Reader (PerkinElmer).

Time-of-addition (ToA) antiviral drug analysis

To determine whether the timing of the compound addition affects the inhibitory effectiveness towards HCMV infection, the drugs ABE, BCV, LDC4297, or P-020 were added at different time points: (i) three days before infection (-3 d) with repeated application on the day of infection; (ii) on the day of infection (d 0); (iii) two days after infection (+2 d); and (iv) two days before infection only (-2 d), without post-infection treatment. A total number of 1.35×10^4 HFF cells/well was seeded in 96-well plates and infected with HCMV AD169-GFP (MOI 0.25-0.5 according to GFP-FU/7 d). At 7 d p.i., cells were fixed and viral GFP reporter signal was measured using a Victor X4 Multilabel Microplate Reader (PerkinElmer, Waltham, MA, USA).

Supplementary Figures

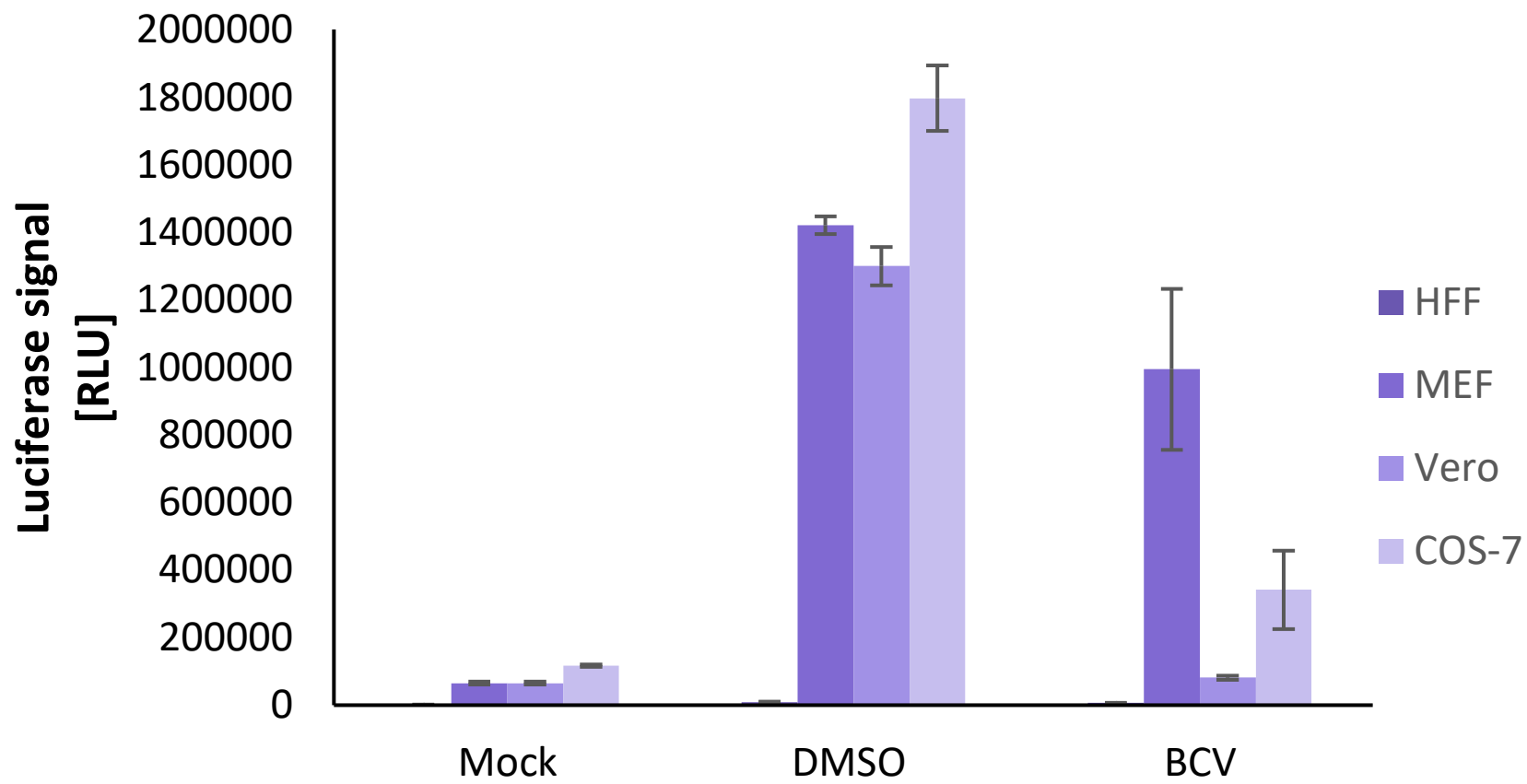

Fig. S1. Cell types supporting the productive replication of MHV-68-Luc. Primary human and murine fibroblasts MEFs and HFFs, as well as immortalized Vero and COS-7 cells were cultivated in the 96-well plate format and used for MHV-68-Luc infection, comparing uninfected (mock) and infected cells under brincidofovir treatment (BCV 62.5 nM versus DMSO control). At 4 d p.i., cells were harvested, and absolute luciferase signals were scored. All values were determined as mean ± SD, as based on determinations in quadruplicate.

Antiviral compounds	Drug-sensitive herpesviruses	Main mode of action/targets	Chemical structures
ABE (6)	HCMV, EBV, MCMV, MDV	host CDK4/6	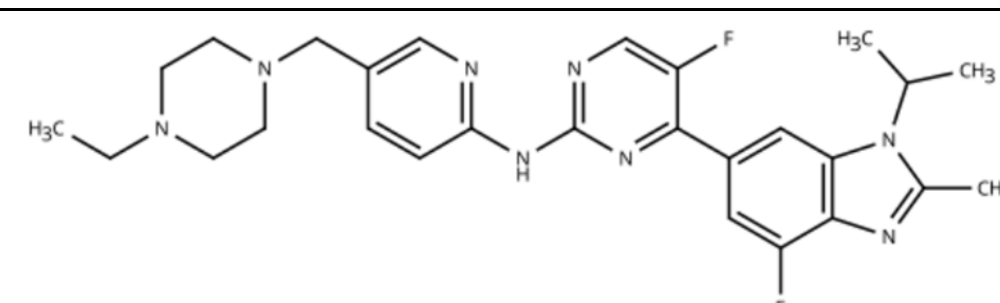
BCV (13)	HSV-1, HSV-2, VZV, HCMV, HHV-6A, HHV-6B, EBV, KSHV	viral DNA polymerase	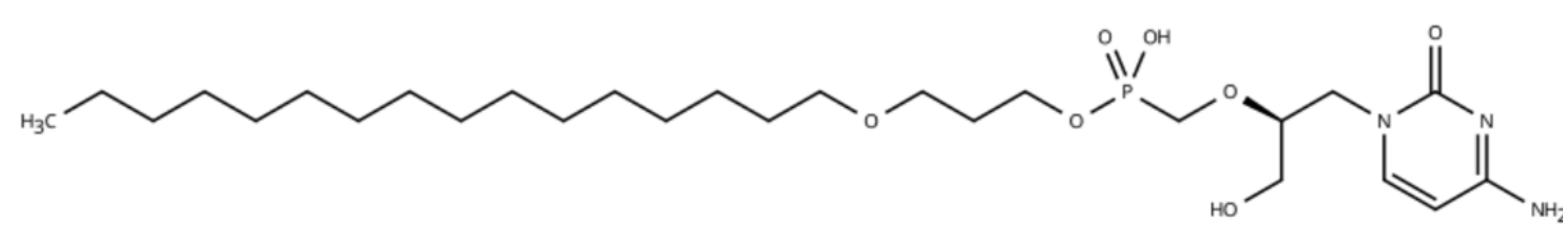
LDC4297 (7)	HSV-1, HSV-2, VZV, HCMV, HHV-6A, GPCMV, MCMV, EBV	host CDK7	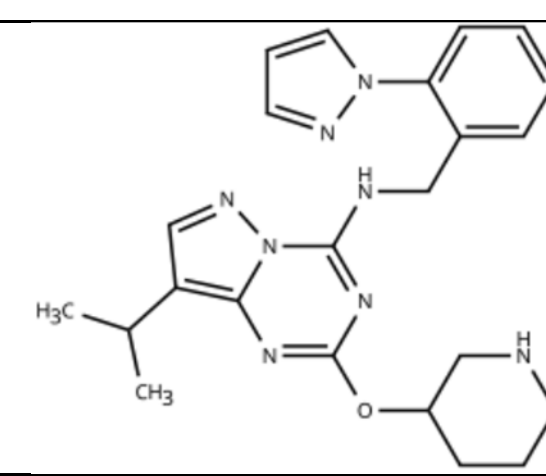
P-020 (8)	HSV-1, VZV, HCMV, ChCMV, RhCMV, EBV, KSHV	viral NEC	unpublished

Fig. S2. Overview of antiviral compounds regarding antiherpesviral activities, known details about mode of action/targets, and chemical structures. Specific characteristics of four analyzed antiviral compounds are depicted, i.e. abemaciclib (ABE, ref. 6, CAS RN 1231929-97-7), brincidofovir (BCV, ref. 13, CAS RN 444805-28-1), LDC4297 (ref. 7, CAS RN 1453834-21-3), and P-020 (chemical structure unpublished, ref. 8). As far as published to date, the chemical structures were retrieved from the PubChem database. Abbreviations: HSV-1, herpes simplex virus 1; HSV-2, herpes simplex virus 2; VZV, varicella zoster virus; MDV, Marek's disease virus; HCMV, human cytomegalovirus; HHV-6A, human herpesvirus 6A; HHV-6B, human herpesvirus 6B; ChCMV, chimpanzee cytomegalovirus; RhCMV, rhesus macaque cytomegalovirus; MCMV, murine cytomegalovirus; GPCMV, guinea pig cytomegalovirus; EBV, Epstein-Barr virus; KSHV, Kaposi sarcoma-associated herpesvirus; CDK, cyclin-dependent kinase; NEC, nuclear egress complex.

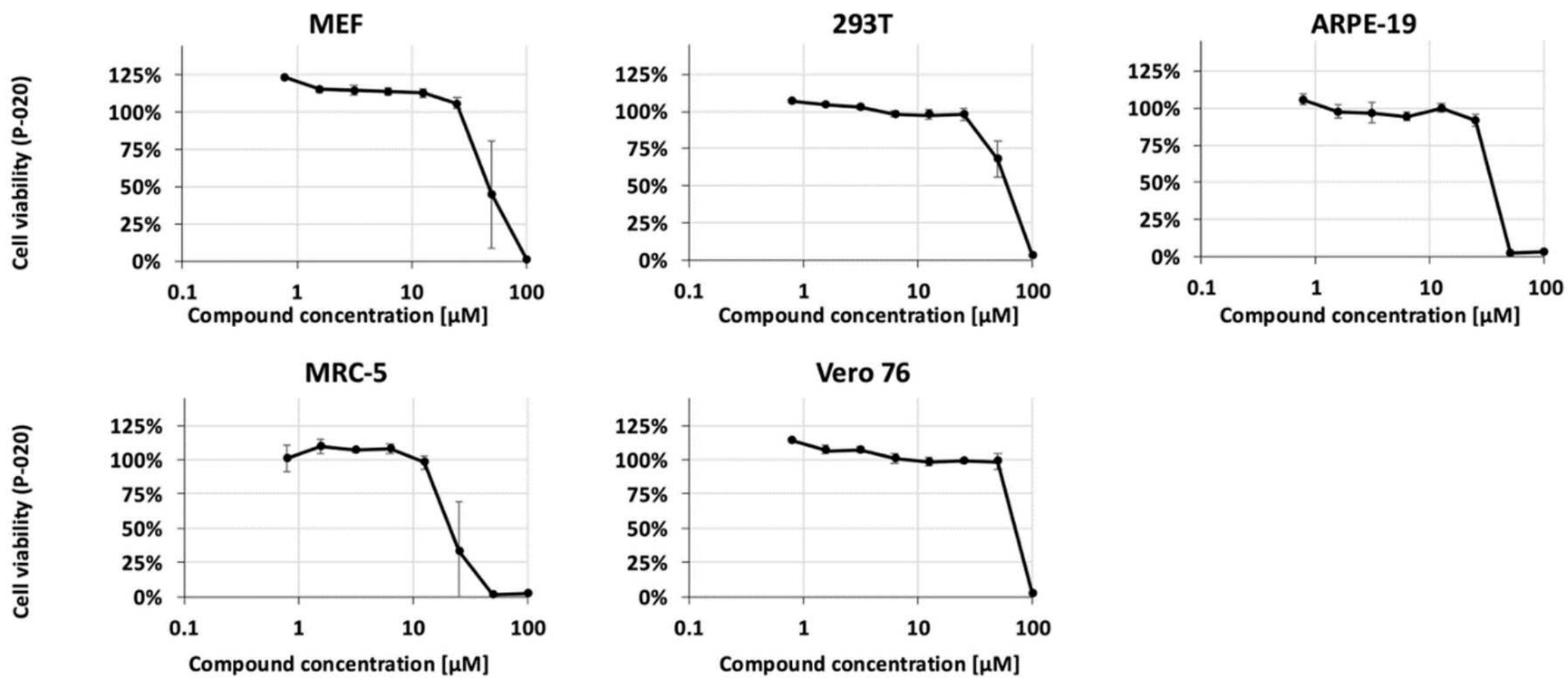

Fig. S3. Levels of cytotoxicity determined for the recently identified pharmacophore compound P-020. Selected cell types were treated with P-020 at a series of concentrations of 0.78, 1.56, 3.13, 6.25, 12.5, 25, 50, and 100 µM. At 5 d post-treatment, cell viability was measured by Neutral Red uptake assay (triplicate determination), and values were expressed as a percentage relative to DMSO.

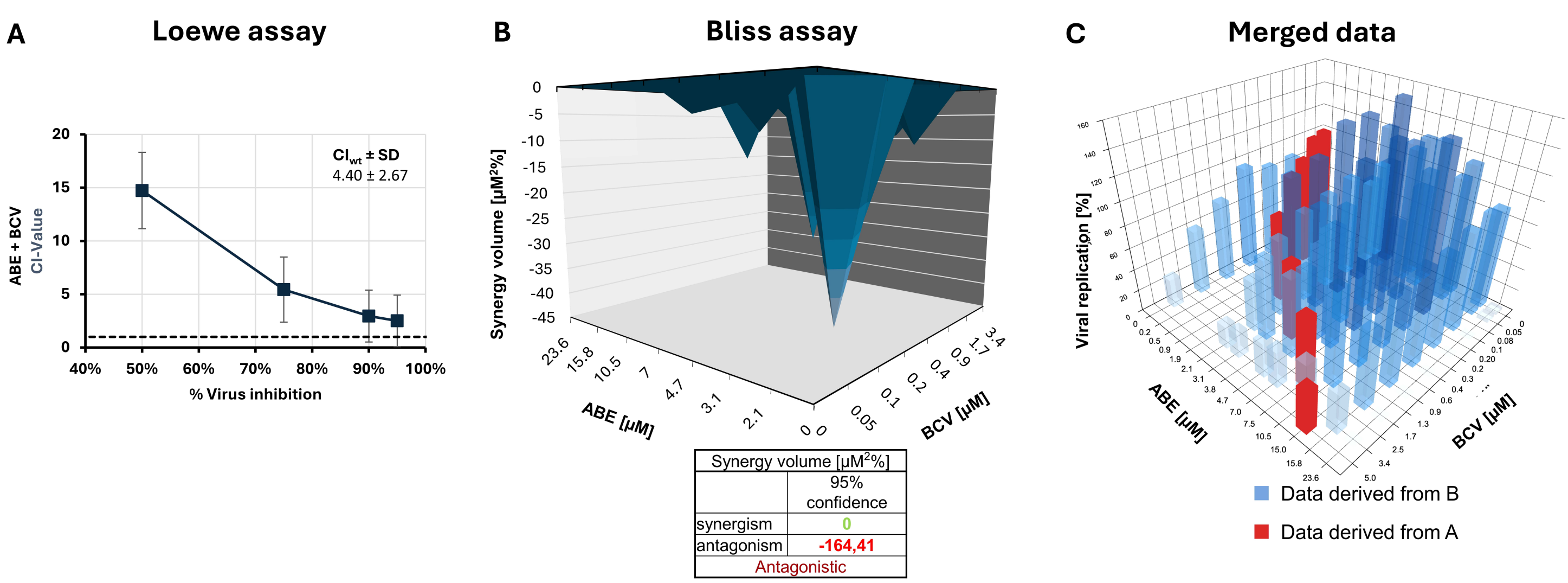

Fig. S4. Synergism interaction analysis of the combined treatment ABE + BCV against HSV-1. **(A)** Loewe additivity fixed-dose analysis (ABE + BCV ratio 4:1). Data are presented as mean weighted CI values (CI_{wt} ± SD), corresponding to the defined values of virus inhibition (50%, 75%, 90%, 95%), collected from n=2 independent experiments. **(B)** Bliss independence checkerboard analysis of the same combination, generated within MacSynergy II. The types of synergy volume [$\mu M^2\%$] values defining the drug-drug interaction were as follows: values below −100, antagonistic; −100 to +50, additive; +50 to +100, moderately synergistic; above +100, strongly synergistic (6, 10, 14). **(C)** Merged data presented in a three-dimensional visualization, combining the raw dose-response data of the Bliss independence checkerboard assay (panel B, blue bars; color-graded according to magnitude of viral replication) and Loewe additivity fixed-dose assay (panel A, solid red bars). This merged representation allows for direct spatial comparison of both assay datasets, while providing a combined representation and direct visual validation of antiviral efficacy. The graphic was generated by the use of RStudio software (available via www.r-studio.com).

Supplementary References

1. Repetto G, del Peso A, Zurita JL. Neutral red uptake assay for the estimation of cell viability/cytotoxicity. Nature protocols. 2008;3(7).
2. Marschall M, Freitag M, Weiler S, Sorg G, Stamminger. Recombinant green fluorescent protein-expressing human cytomegalovirus as a tool for screening antiviral agents. Antimicrobial agents and chemotherapy. 2000;44(6).
3. Briestenská K, Šamšulová V, Poláková M, Mistríková J. Recombinant luciferase-expressing murine gammaherpesvirus 68 as a tool for rapid antiviral screening. Acta virologica. 2019;63(4).
4. Elliott G, O'Hare P. Live-cell analysis of a green fluorescent protein-tagged herpes simplex virus infection. Journal of virology. 1999;73(5).
5. Obergfäll D, Wiebusch K, Markus W, Mona S, Malena Barillas D, Jintawee K, et al. Cyclin-Dependent Kinase 8 Represents a Positive Regulator of Cytomegalovirus Replication and a Novel Host Target for Antiviral Strategies. Pharmaceutics. 2024;16(9):1238.
6. Wild M, Kicuntod J, Seyler L, Wangen C, Bertzbach LD, Conradie AM, et al. Combinatorial Drug Treatments Reveal Promising Anticytomegaloviral Profiles for Clinically Relevant Pharmaceutical Kinase Inhibitors (PKIs). International Journal of Molecular Sciences. 2021;22(2):575.
7. Hutterer C, Eickhoff J, Milbradt J, Korn K, Zeitträger I, Bahsi H, et al. A novel CDK7 inhibitor of the Pyrazolotriazine class exerts broad-spectrum antiviral activity at nanomolar concentrations. Antimicrobial agents and chemotherapy. 2015;59(4).
8. Tillmanns J, Battisti V, Kicuntod J, Hahn F, Obergfäll D, Geiger P, et al. The conserved core nuclear egress complex (NEC) as an antiherpesviral drug target: Pharmacophore-based identification of NEC-specific inhibitors. Antiviral research. 2025;239.
9. Chou TC, Martin N. CompuSynFor Drug Combinations User’s Guide: A Computer Program for Quantitation of Synergism and Antagonism in Drug Combinations, and the Determination of IC 50 and ED50 Values. 2005.
10. Chou TC. Theoretical basis, experimental design, and computerized simulation of synergism and antagonism in drug combination studies. Pharmacological reviews. 2006;58(3).
11. Wild M, Karner D, Eickhoff J, Wagner S, Kicuntod J, Chang W, et al. Combined Treatment with Host-Directed and Anticytomegaloviral Kinase Inhibitors: Mechanisms, Synergisms and Drug Resistance Barriers. Pharmaceutics. 2023;15(12).
12. Prichard MN, Shipman C. A three-dimensional model to analyze drug-drug interactions. Antiviral research. 1990;14(4-5).
13. Chemaly RF, Hill JA, Voigt S, Peggs KS. In vitro comparison of currently available and investigational antiviral agents against pathogenic human double-stranded DNA viruses: A systematic literature review. Antiviral Research. 2019;163:50–8.
14. Wild M, Hahn F, Brückner N, Schütz M, Wangen C, Wagner S, et al. Cyclin-Dependent Kinases (CDKs) and the Human Cytomegalovirus-Encoded CDK Ortholog pUL97 Represent Highly Attractive Targets for Synergistic Drug Combinations. Int J Mol Sci. 2022;23(5).
